# Childhood socio-emotional and cognitive development and adolescents NEET (not in education, employment or training): findings from the UK Millennium Cohort Study

**DOI:** 10.1136/bmjopen-2025-109720

**Published:** 2026-06-22

**Authors:** Lateef Akanni, Michelle Black, Kalu Udu, Yanhua Chen, Rosalie Cattermore, Oluwaseun B Esan, Hanna Creese, G J Melendez-Torres, Dougal Hargreaves, Nicholas Kofi Adjei, David Taylor-Robinson

**Affiliations:** 1 School of Social and Political Sciences, University of Glasgow, Glasgow, Scotland, UK; 2 Department of Public Health, Policy and Systems, University of Liverpool, Liverpool, UK; 3 School of Public Health, Imperial College London, London, UK; 4 University of Exeter, Exeter, UK

**Keywords:** Cognition, Behavior, EDUCATION & TRAINING (see Medical Education & Training), Longitudinal studies, Child & adolescent psychiatry

## Abstract

**Abstract:**

**Background:**

There is a growing concern about the increasing number of young people who are not in employment, education or training (NEET) globally. This study investigates the impact of concurrent cognitive and socio-emotional development trajectories in childhood on NEET status in adolescence in a UK cohort.

**Method:**

We analysed longitudinal data on 8368 children from the UK Millennium Cohort Study. Exposure trajectories of cognitive and socio-emotional development from age 3 to 14 years were characterised using group-based multi-trajectory models. We used Poisson regression to examine associations between developmental trajectories and NEET status at age 17, adjusting for confounders. Population-attributable fractions were estimated to quantify NEET proportions attributable to the developmental problems.

**Results:**

At age 17, 3.5% of participants were NEET, of which about one-third (38%) were not economically active. Children with persistent cognitive and socio-emotional development problems had a fourfold increased risk of being NEET (adjusted risk ratio (ARR) 3.5; 95% CI 2.3 to 5.3), and those with late socio-emotional problems had a threefold increased risk (3.0; 95% CI 2.1 to 4.3), compared with children in the no problem group. Early and resolving socio-emotional and cognitive problems were not associated with being NEET. An estimated 28% (95% CI 18% to 36%) of NEET cases were attributable to cognitive and socio-emotional behaviour problems in childhood.

**Conclusion:**

Childhood cognitive and socio-emotional development plays a critical role in shaping pathways to education and employment in adolescence. Policies and strategies aiming to reduce NEET should target early social and emotional skills alongside efforts to support academic achievement.

**STRENGTHS AND LIMITATIONS OF THIS STUDY**

- The study uses longitudinal data from a contemporary and representative cohort of UK children.
- The study combines measures of cognitive ability and socio-emotional behaviour during childhood and evaluates the joint effects on youth not in education, employment or training (NEET) status.
- A major limitation was the inability to capture transitions in the NEET status as it was measured at a single time point.
- As with most longitudinal cohort studies, missing data is inevitable and hence a challenge for analysis.

## Introduction

 Education, training and employment are key gateways to human capital development, shaping long-term health, well-being and economic prosperity.^1^ These domains are particularly critical during the transition from adolescence to adulthood, a developmental period in which pathways are often set for future life chances. The category of young people not in education, employment or training (NEET) is used internationally as a marker of youth disengagement and lost productivity. Globally, NEET incidence has been increasing yearly, with 20.4% of young people aged 15–24 years NEET in 2024;^2^ with a corresponding estimate of 13.6% among 16–24 year olds in the UK.^3^

The long-term consequences of youth NEET are well established, including poorer health outcomes, lower lifetime earnings and broader economic costs to society.^4^,^6^ A wide range of risk factors, including individual, familial, societal and structural factors such as poverty, have been shown to increase the likelihood of becoming NEET.^7^ Mental health issues and psychosocial problems in adolescence are also consistently associated with increased risk of NEET.^6 8 9^ Structural barriers such as social exclusion and labour market demands further constrain access to opportunities, disproportionately affecting the most disadvantaged, particularly those with pre-existing mental health difficulties.^7 10^ These risk factors often interact and accumulate over the life course, suggesting that meaningful policy responses on NEET should recognise both individual and structural determinants.

Emerging evidence suggests that individual risk factors for NEET may start in early childhood. For instance, lack of school readiness at age 4–5 in England has been linked to later NEET, largely but not solely through academic attainment.^11^ School readiness also incorporates social and emotional development, key labour market skills,^12^ which are increasingly recognised as core skills for labour market success,^13^ independent of cognitive ability. Thus, disentangling the contributions of these developmental issues could help inform the type and timing of interventions throughout children’s development, beyond academic attainment, which may impact NEET status.

Previous studies have shown that patterns of cognitive and socio-emotional development across childhood may impact health and educational outcomes in adolescence,^14^,^17^ with the timing of emergence of problems to be a crucial factor. For example, our previous research has shown that children with early socio-emotional and cognitive problems, where socio-emotional problems resolve and cognitive problems improve from age 3–14 years did not experience adverse health outcomes in adolescence^14^ but remained at increased risk of poor educational attainment at age 16.^15^ This distinction in the impact of early and resolved behavioural problems (and reducing cognitive problems), which is positive for health but negative for education, provides an opportunity to understand whether good social and emotional skills (despite some cognitive problems) are also protective for not becoming NEET. Furthermore, quantifying associations between trajectories of socio-emotional and cognitive development, which emerge early, later or are persistent, and NEET status provides an opportunity to better understand the balance between the impact and timing of problems in socio-emotional and cognitive development on NEET status. Therefore, this study aimed to assess the impact of concurrent cognitive and socio-emotional development trajectories in childhood on NEET status at age 17 years in a UK cohort.

## Method

### Study settings and participants

We used longitudinal data from the UK Millennium Cohort Study (MCS). MCS is a large-scale, population-based cohort study tracking over 18 000 children born in the UK between September 2000 and January 2002, with follow-up at ages 3, 5, 7, 11, 14 and 17 years, corresponding to waves 1 to 7. The number of responding families at the different waves was 18 552 (wave 1), 15 590 (wave 2), 15 246 (wave 3), 13 857 (wave 4), 13 287 (wave 5), 11 726 (wave 6) and 10 625 (wave 7). MCS collects information on various topics at each wave, including education, employment, health, family socio-economic circumstances and structure, cohort members’ (CMs) behavioural and cognitive development. Information was usually provided by the primary caregiver, mostly the CMs’ mother. However, parental involvement was minimal in wave 7 when the child was 17 years old. Detailed information on the survey design, sampling and the scope of the MCS is detailed elsewhere.^18^ This study is reported in accordance with the Strengthening the Reporting of Observational Studies in Epidemiology (STROBE) guidelines (see online supplemental table S2).

### Exposures

Our main exposures were trajectories of cognitive and socio-emotional development from early childhood to adolescence (age 3 to 14 years) as previously defined in Black *et al*.^14^ Four trajectory groups of child cognitive and socio-emotional development experienced by children in the UK Millennium Cohort were identified using a group-based multi-trajectory modelling approach.^19^ Socio-emotional behaviour was measured using the Strengths and Difficulties Questionnaire,^20^ completed by the parent, while cognitive development was measured from the results of standard cognition tests administered individually to CMs at ages 3, 5, 7, 11 and 14 years (the list and description of measures are summarised in online supplemental appendix). The four trajectory groups, based on predicted probabilities, were ‘no problems’ (76.5%); ‘early and resolving cognitive and socio-emotional problems’ (8.6%); ‘late socio-emotional problems’ (10.1%) and ‘persistent cognitive and socio-emotional problems’ (4.8%) (see online supplemental figure S1).

### Outcomes

The primary outcome was NEET status at age 17. We measure NEET using a binary construct from responses to four questions related to NEET status. CMs were asked the following questions in wave 7 of the MCS: (1) “Are you currently going to school or college?”, (2) “Are you currently doing an Apprenticeship?”, (3) “Are you currently doing any kind of traineeship, training course or scheme?” and (4) “Are you currently doing any kind of paid job? We categorised participants as ‘NEET’ (coded 1), if they responded “no” to all the four questions, and those who responded “yes” in an any of the four questions were classified as ‘Not NEET’ (coded 0).

The secondary outcome was economically inactive NEET. Young people who were NEET may be either unemployed and actively looking for work or economically inactive. Economic inactivity was defined as not being in education, employment or training, and not actively looking for work in the 4 weeks prior to the interview.^3^ This was based on responses to two questions regarding current job search activity and any attempt to find a paid job, including both being an employee or being self-employed, within the past 4 weeks.^21^ Participants who were NEET and reported no active job search during this period were classified as economically inactive NEET (coded as 1) and otherwise coded as 0.

### Confounders

**Figure 1:**
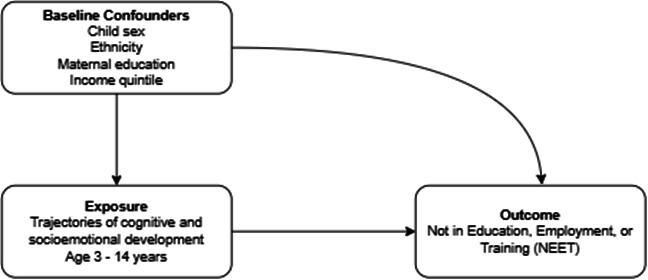
Directed Acyclic Graph for the study.

We adjusted for potential confounders associated with child development in early life and NEET, guided by a directed acyclic graph (figure 1). The key confounders considered include child sex, maternal education level (degree or higher, diploma, A levels, GCSE A-C, GCSE D-G or no qualifications), maternal ethnicity (white, mixed, Indian, Pakistani and Bangladeshi, Black or Black British or other ethnic groups), and income quintile at baseline.

### Statistical analysis

We first described the distribution of sample characteristics including exposures, confounders and outcomes across the trajectories of cognitive and socio-emotional behaviour using descriptive statistics. We then estimated Poisson regression models to examine the association between the identified trajectory groups and NEET status at age 17. Two models were constructed: unadjusted model and model adjusted for confounders. We generated risk ratios with robust standard errors comparing each developmental trajectory group to “no problem” trajectory group. All estimates were reported with 95% CIs which provide information about both the magnitude and precision of the estimated association. Estimates were weighted using longitudinal weights to account for attrition, non-response and sampling design. To assess the potential population-level impact of developmental trajectories, we calculated population attributable fractions (PAFs),^22^ which estimate the proportion of NEET cases that could be prevented if exposure to cognitive and socio-emotional behavioural problems were reduced to the same levels as youth in no problems. We further conducted two robustness checks. First, to account for uncertainty in trajectory group membership, we applied Vermunt18 three-step approach.^23^ Second, we addressed potential bias due to missing covariate data by using multiple imputation by chained equations (MICE) (30 imputed data sets), with results pooled using Rubin’s rules. Third, we evaluated the results’ sensitivity to unmeasured confounding by calculating ‘E-values’ for each exposure contrast using the estimated ORs and CIs.^24^ Statistical analyses were performed using Stata (V.18.5).

## Results

**Figure 2:**
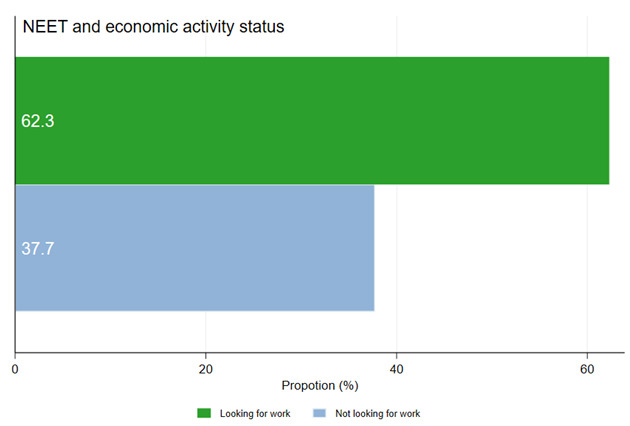
Proportion of NEET in Millennium Cohort Study by job search status. NEET, not in education, employment or training.

There were 10 625 participants in the MCS when the CMs were aged 17 years. We included a total of 8368 CMs in the analysis after excluding those with missing data on the exposure trajectories (online supplemental figure S2). The overall prevalence of NEET at age 17 was 3.5% (289 CMs). Among those classified as NEET, 62% (n=180) were economically active, that is, looking for or available for work during the interview period, while 38% (n=109) were not economically active (figure 2). Table 1 shows the characteristics of the CMs by NEET status. Among participants in the “no problem” trajectory group, NEET prevalence was 2.4%. In contrast, NEET status was more prevalent among those with developmental problems: 13% in the persistent cognitive and socio-emotional problems trajectory group, 8% in the late socio-emotional problems and 4% in the early cognitive and socio-emotional problems group. There was a social gradient to NEET status based on household income quintile and maternal educational qualification. The higher the income quintile and education level, the lower the prevalence of NEET.

**Table 1:** Baseline characteristics and outcome by NEET, observed data

	No NEET (n=8079)	NEET (n=289)
Trajectories of socio-emotional and cognitive development		
No problems	6465 (97.6)	160 (2.4)
Early and resolving cognitive and socio-emotional problems	645 (95.6)	30 (4.4)
Late socio-emotional problems	684 (92.2)	58 (7.8)
Persistent cognitive and socio-emotional problems	285 (87.4)	41 (12.6)
Child’s sex		
Male	3717 (96.6)	131 (3.4)
Female	4087 (96.6)	143 (3.4)
Missing	275 (94.8)	15 (5.2)
Maternal ethnicity		
White	6414 (96.5)	236 (3.5)
Mixed	76 (93.8)	5 (6.2)
Indian	241 (99.2)	2 (0.8)
Pakistani and Bangladeshi	640 (97.0)	20 (3.0)
Black or Black British	262 (97.4)	7 (2.6)
Other ethnic group	156 (98.7)	2 (1.3)
Missing	290 (94.5)	17 (5.5)
Maternal education		
Degree plus	1820 (99.3)	13 (0.7)
Diploma	773 (97.5)	20 (2.5)
A-levels	841 (97.2)	24 (2.8)
GCSE A-C	2408 (96.0)	100 (4.0)
GCSE D-G	645 (95.0)	34 (5.0)
None	1304 (94.2)	81 (5.8)
Missing	288 (94.4)	17 (5.6)
Household income quintile		
Lowest quintile	1337 (93.3)	96 (6.7)
Second quintile	1478 (94.4)	88 (5.6)
Third quintile	1490 (96.9)	48 (3.1)
Fourth quintile	1666 (98.5)	25 (1.5)
Highest quintile	1810 (99.2)	15 (0.8)
Missing	298 (94.6)	17 (5.4)

NEET, not in education, employment or training.

**Figure 3:**
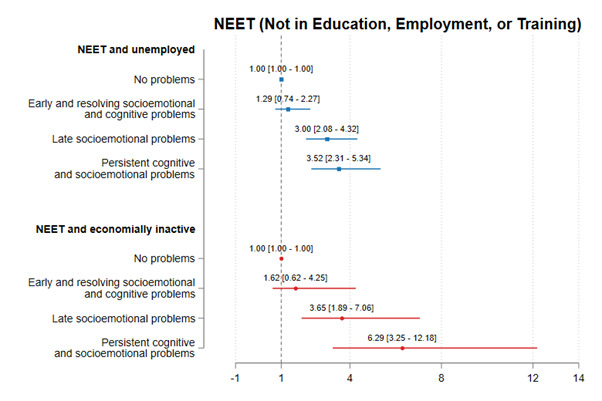
Adjusted risk ratios of predicted developmental trajectories and being NEET at age 17 years in the UK Millennium Cohort Study.

We summarise the estimated association between developmental trajectory groups and NEET status in table 2 and figure 3. The unadjusted and adjusted models both indicate that exposures to early problems were not significantly associated with being NEET at age 17, compared with children in the ‘no developmental problems’ group. In contrast, adolescents with late socio-emotional problems were three times more likely to be NEET (adjusted risk ratio (ARR) 3.00, 95% CI 2.08 to 4.32) and those with persistent cognitive and socio-emotional problems have three and a half times increased risks of being NEET compared with the no problems group (ARR 3.52, 95% CI 2.31 to 5.34).

**Table 2:** Risk ratios between multi-development trajectories and adolescents’ NEET in the UK Millennium Cohort Study

Outcome	Model 1		Model 2	
	Estimate	PAF	Estimate	PAF
**NEET and unemployed**				
Trajectories of socio-emotional and cognitive development (ref: *no problems*)				
Early and resolving cognitive and socio-emotional problems	2.44 (1.54–3.88)	5% (1%–9%)	1.29 (0.74–2.27)	2% (−2% to 5%)
Late socio-emotional problems	4.10 (2.92–5.76)	18% (11%–24%)	3.00 (2.08–4.32)	16% (9%–23%)
Persistent cognitive and socio-emotional problems	6.28 (4.27–9.23)	12% (7%–16%)	3.52 (2.31–5.34)	10% (5%–15%)
Total PAF		34% (26%–42%)		28% (18%–36%)
**NEET and not looking for work**				
Trajectories of socio-emotional and cognitive development (ref: *no problems*)				
Early and resolving cognitive and socio-emotional problems	2.97 (1.43–6.16)	6% (0.1%–12%)	1.62 (0.62–4.25)	3% (−4% to 10%)
Late socio-emotional problems	4.56 (2.57–8.11)	18% (7%–28%)	3.65 (1.89–7.06)	17% (6%–23%)
Persistent cognitive and socio-emotional problems	9.44 (5.18–17.17)	16% (7%–24%)	6.29 (3.25–12.18)	15% (6%–23%)
Total PAF		40% (25%–52%)		34% (16%–48%)

Note: Model 1 is the baseline model, and Model 2 adjusts for confounders including child sex, maternal ethnicity, education, and income quintile. The PAF column shows the proportional reductions in cohort members who did not experience NEET if they experienced no developmental problems. The PAF is calculated by comparing two scenarios: scenario 1 (a hypothetical scenario in which all children were in the no problem trajectory) with scenario 0 (the real world in which there are children in the no problem group and other trajectories). The reference group is the no-problem trajectory group.

NEET, not in education, employment or training; PAF, population attributable fraction.

Similar patterns were also observed for the secondary outcome (being NEET and economically inactive). Compared with those in the no-problem group, the relative risks were more than three times higher for the late socio-emotional problems group (ARR 3.65; 95% CI 1.89 to 7.06) and six times higher for the persistent cognitive and socio-emotional behaviour problems group (ARR 6.29; 95% CI 3.25 to 12.18). Estimates were attenuated after adjusting for confounders. However, sequential adjustment for each confounder indicated that income quintile at baseline accounted for the largest proportion of the association between NEET status and developmental trajectories, with maternal education, an indication of early life socio-economic conditions, contributing further. The residual association after controlling for these early-life socio-economic factors suggests that NEET status at adolescence captures additional risk not explained by early-life disadvantage. (see online supplemental figure S5).

The estimated PAFs, which quantifies the proportion of NEET cases attributable to each developmental trajectory, is presented in table 2. Assuming a cause–effect relationship, the adjusted PAF shows that approximately 28% (95% CI 18% to 36%) of NEET cases could be attributed to cognitive and socio-emotional behaviour problems during childhood. The proportion was higher for adolescents who were NEET and economically inactive, with an adjusted PAF of 34% (95% CI 16% to 48%). The sensitivity analyses, including Vermont three-steps for classification uncertainty and multiple imputation to address missing covariate data, show similar results to the main estimation (see online supplemental figures S3 and S4). Additionally, the E-values indicated that the estimated associations for late and persistent cognitive and socio-emotional problems were robust to unmeasured confounding but not for early and resolving cognitive and socio-emotional problems (see online supplemental table S1).

## Discussion

We used the MCS, a large nationally representative cohort sample of UK children, to show the association between joint trajectories of socio-emotional and cognitive development throughout childhood and the likelihood of not being in education, employment or training in early adolescent years. We found that 3.5% of participants were NEET at age 17, consistent with the national estimate at 3.6% for 16–17 years olds in 2017 when CM were 17 years.^25^ We further showed that approximately 28% of NEET cases were attributable to adverse cognitive and socio-emotional development in childhood, if causality is assumed.

Our findings suggest that addressing cognitive and socio-emotional behaviour problems during childhood may substantially reduce the risk of becoming NEET in adolescence. Indeed, the timing and persistence of developmental problems were found to be critical. Children with late-onset or persistent problems were at higher risk of NEET, while those with early problems that were on a resolving trajectory did not significantly increase risk compared with children in the ‘no problems’ group.

The distinct role of socio-emotional behaviour and its relationship with NEET warrants further investigation. Our results highlight that children who start school with socio-emotional behaviour problems and cognitive problems but are on a resolving trajectory, with behaviour problems resolving by age seven and cognitive problems reducing, are not associated with NEET status. We know from our other studies that this trajectory group is associated with poor educational attainment at age 16.^15^ So, it appears that resolving socio-emotional behaviour problems within the first few years of starting school may be protective of later adverse health and NEET, despite a higher risk of poor educational attainment. This implies that reducing NEET is not limited to exam results but about fostering the social and emotional well-being that is necessary for labour market engagement. Further research could help to unpick possible protective mechanisms, building on the mediating mechanism of socialisation through peers, schools and family, to better understand how social and emotional skills in childhood forecast skills in late adolescence/adulthood for labour market success.^26^

In relation to the development of socio-emotional behaviour problems in late childhood/early adolescence, we see over a threefold increase in the risk of both NEET status and being NEET and not economically active. This reflects findings in relation to conduct and emotional problems which have been shown in UK cohorts, from 1958 and 1970, to be associated with increased odds of economic inactivity, more so for conduct problems.^27 28^ Understanding how to better support these aspects of development is crucial if we are to address the increasing prevalence of economic inactivity in working-age people.^29^ Education, employment and training in young people are precursors for working-age economic activity. The current UK government recognises this, and one of its missions is to break down barriers to opportunity by expanding high-quality education, employment and training to ensure more people are on pathways to good prospects in the next decade.^30^ Social mobility policy should also focus on improving routes to employment via apprenticeships and incentivising workplaces to continue with skills development for young people, working across geographical and organisational boundaries and with young people themselves.^31^ Our analysis contributes to understanding the barriers to achieving this goal. Although further research is needed to better understand the mediating pathways, our findings provide strong evidence that NEET status and economic inactivity in adolescence are closely linked to earlier cognitive and socio-emotional development. Policy efforts need to recognise the importance of developing capabilities and prioritise early intervention strategies that address behavioural and developmental challenges, particularly those emerging or persisting into later childhood.^32^

Implications beyond social mobility relate to schools and health. Early years investment is crucial to help children’s school readiness, and for those not ready at school entry, schools should be resourced to support development early, ensuring children do not continue to fall behind. The UK government’s focus on school readiness (75% of children should be school-ready by 2028) is therefore a welcome and evidence-based policy initiative. Our findings suggest that the emphasis on social and emotional well-being in schools is key, with potential benefits for health, employment and welfare. Poor social and emotional well-being in childhood is a precursor for poor mental health in later life. If we are to reduce the burden of mental ill health in working-age people^33^ - a key factor in the rising numbers of young people claiming disability benefits with associated welfare costs spiralling from £36 billion in 2019–2020 to £48 billion in 2023–2024,^34^ then we need to act on social and emotional well-being early in life and throughout the child-adolescent life course. This requires a cross-sectoral approach where the health and education sectors work together to support children’s developmental needs,^35^

To be effective, cross-sector health and education policy needs to be integrated with economic and social policies aimed at mitigating structural disadvantage, in recognition of the contextual factors, including increasing child poverty, which impact student experience, well-being, attendance and attainment. This study adds to a growing body of evidence linking early developmental trajectories to later educational and economic outcomes.^6 8^ Previous research also shows a strong social patterning in developmental pathways, with children from socio-economically disadvantaged backgrounds more likely to follow adverse trajectories.^14 36^ Our analyses show that the association between adverse child development and NEET in adolescence persists after adjusting for key measures of disadvantage, suggesting an independent developmental impact. Therefore, it is imperative to address inadequacies and inequalities in schools’ funding,^37^ so that the opportunity to mitigate against disadvantage through the development of capabilities in school is not missed.

The study has some limitations. NEET status was measured at a single time point, which may not capture the dynamic nature of youth disengagement or transitions from compulsory education into further education, training or employment. Although this is a common limitation,^38^ NEET status at age 17 is of particular relevance given the potential long-term implications for health and employment.^39^ Future studies using longitudinal and repeated measures of NEET are needed to distinguish temporary from longer-term disengagement.

Also, our analysis did not capture the causality of the relationships between developmental trajectory and NEET. Hence, more causally informed analyses are needed to better understand these relationships. As with any longitudinal study, the temporal delay between the analysis of the cohort (age 17 in 2017) and the present day may limit what we can infer for policy relevance. While we have shown that the prevalence of NEET in 2025 is similar to that of 2017, in that time we have had a global pandemic with lasting impact on the mental health and well-being of young people, which may mean that policy interventions to reduce the number of young people who are NEET today may need to have a specific focus on mental health. Finally, attrition and missingness are major concerns in longitudinal studies as they pose threats to data quality and could result in estimate bias and underestimation of the observed associations. Nonetheless, we conducted sensitivity analyses using multiple imputations to address missing data. Our findings are further strengthened using a large contemporary UK birth cohort, enhancing the generalisability and policy relevance of the result. Currently, the England Raising Participation Age legislation, passed in 2013, mandates the participation of young people aged 16–17 years in education, training or employment, recognising the importance of early engagement in these activities.

## Conclusion

Using a representative birth cohort of UK children, we show that adverse cognitive and socio-emotional development, which persists and socio-emotional problems which emerge late in childhood are strongly associated with increased likelihood of being NEET in adolescence. Coordinated cross-sectoral actions, including health, education and economic systems, are needed to address developmental problems. Interventions that target cognitive and socio-emotional skills in childhood, particularly in the early years of primary school, may be an effective strategy for reducing the prevalence of NEET and promoting long-term social and economic inclusion.

## Supplementary material

10.1136/bmjopen-2025-109720 online supplemental file 1

## Data Availability

Data are available in a public, open access repository.
