# Supplementary material for: Childhood socio-emotional and cognitive development and adolescents NEET (not in education, employment or training): findings from the UK Millennium Cohort Study
Source: BMJ Open. 2026 Jun 22;16(6):e109720. doi: 10.1136/bmjopen-2025-109720 (PMC13288871; doi:10.1136/bmjopen-2025-109720)
Supplement: online supplemental file 1 [file bmjopen-16-6-s001.docx]

## **Supplementary file**

**Cognitive assessments used at each MCS wave, ages 3-14 years:**

- **MCS2 (Age 3) and MCS3 (Age 5),** BAS Naming Vocabulary: measures expressive verbal ability
- **MCS4 (Age 7),** BAS Word Reading: measures reading ability
- **MCS5 (Age 11),** BAS Verbal Similarities: measures verbal reasoning and verbal knowledge
- **MCS6 (Age 14),** Word Activity Test (subset of the vocab assessment in the 1970 British cohort study survey): measures verbal vocabulary

**
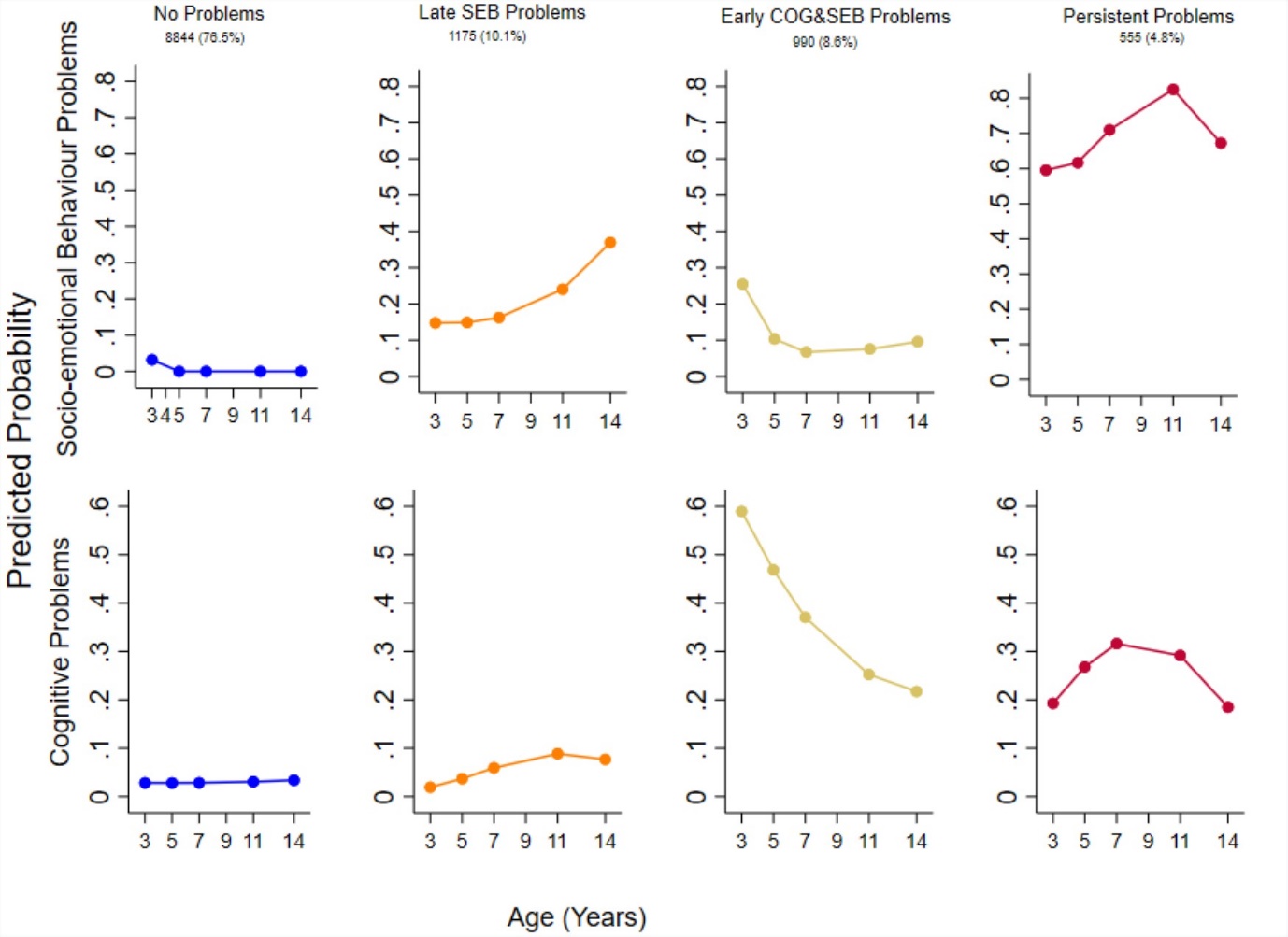
**

Figure S 1: Developmental trajectory groups by age

Note: the figure depicts the Predicted probability of socioemotional behaviour problems and cognitive problems by age and trajectory group in the Millennium Cohort Study. COG, cognitive development; SEB, socioemotional behavioural development. (source: https://doi.org/10.1016/j.jpeds.2023.113611).


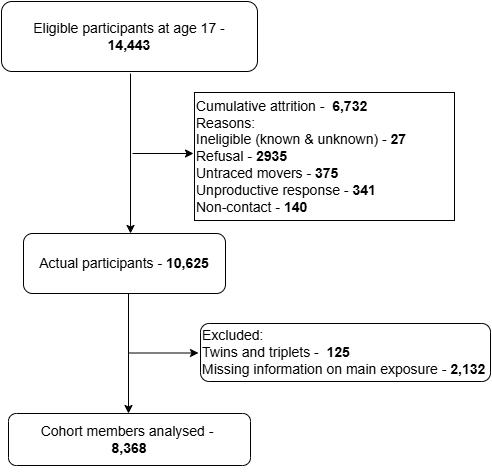


Figure S 2: Study flow diagram showing inclusion and exclusion of cohort participants.


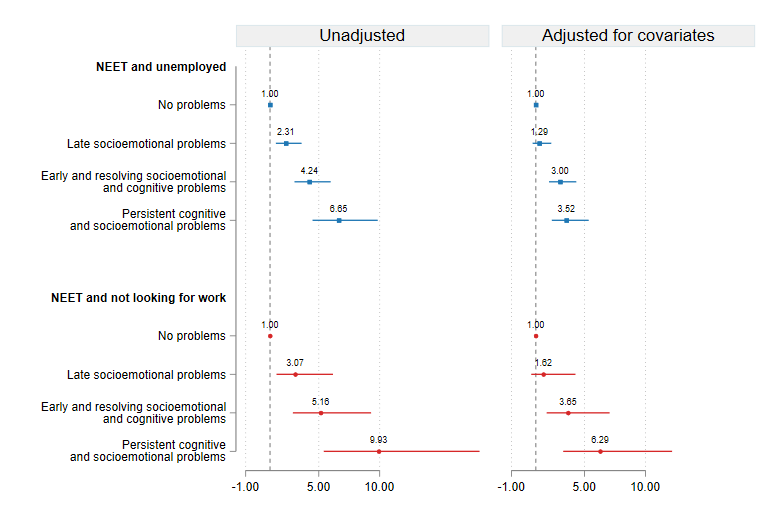


Figure S 3: Trajectory class uncertainties


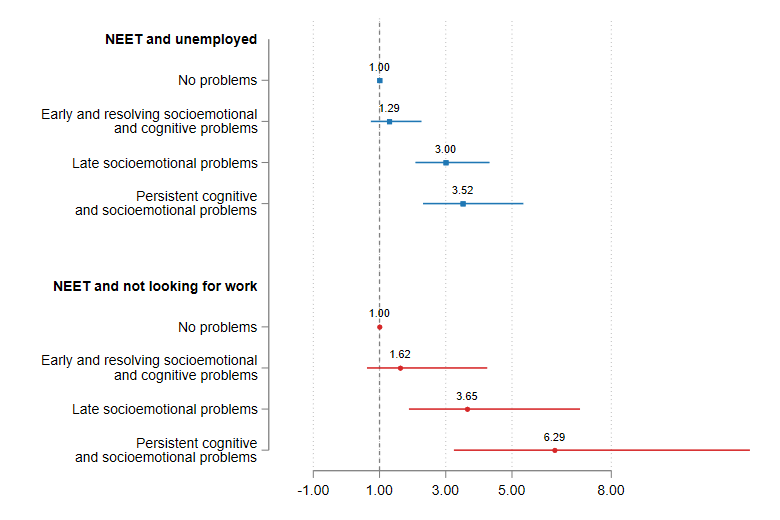


Figure S 4: Multiple imputation results

Table S 1: E-values for unmeasured confounders

| Ref: *No problems* | OR | RR E-value | CI E-value |
| --- | --- | --- | --- |
|  |  |  |  |
| Early and resolving cognitive and socioemotional problems | 1.29 | 1.93 | 1.00 |
| Late socioemotional problems | 3.00 | 5.98 | 3.81 |
| Persistent cognitive and socioemotional problems | 3.52 | 7.38 | 4.38 |

Note: The Table provides the calculated E-values to assess the robustness of observed associations to potential unmeasured confounding. As NEET prevalence was below 10%, odds ratios were interpreted as approximations to risk ratios when deriving E-values. The associations for late socioemotional and persistent cognitive and socioemotional problems show that relatively strong unmeasured confounding would be required to fully explain the observed estimates. In contrast, the association for early and resolving problems showed limited robustness, suggesting that weaker unmeasured confounding could account for the observed association.


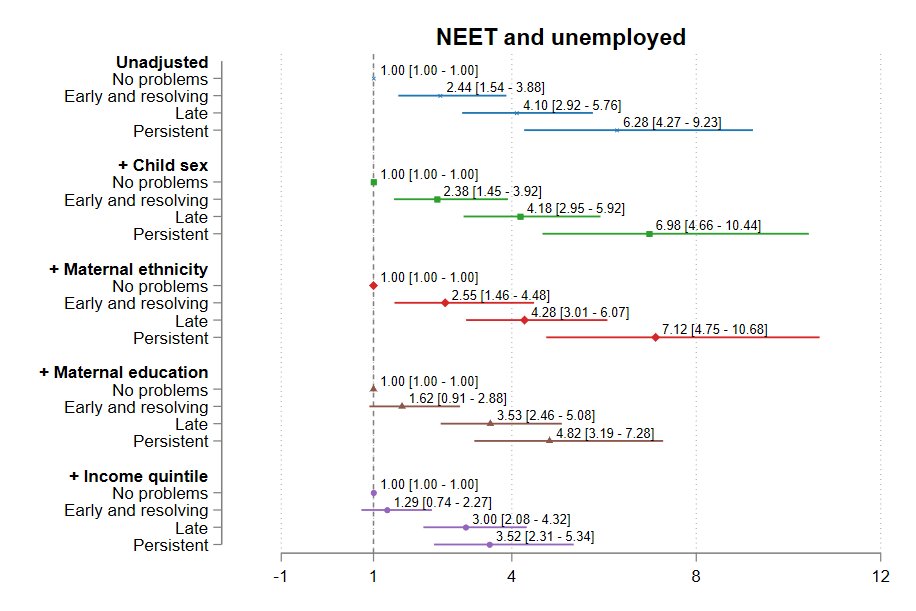

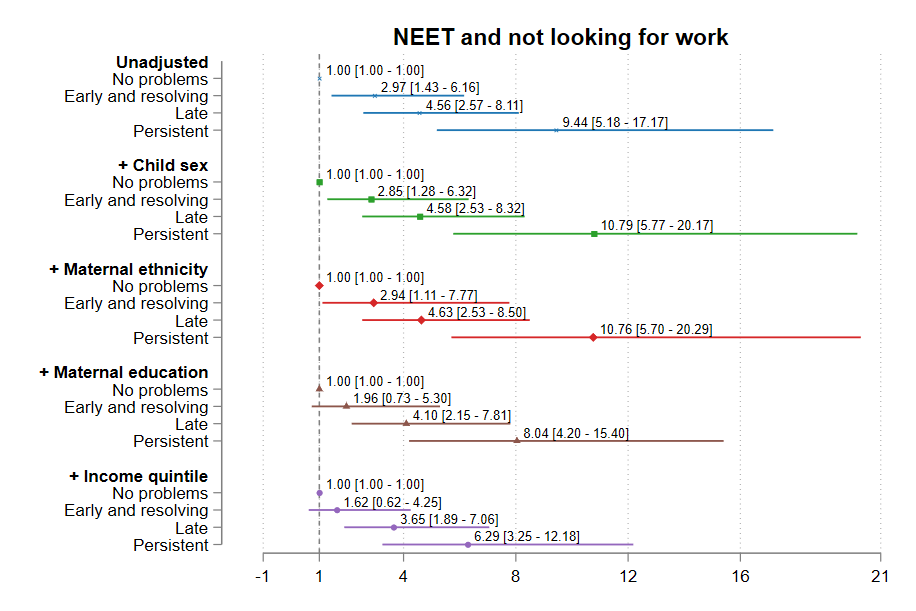


Figure S 5: Sequential adjustment for confounders

Table S 2: Checklist. STROBE Statement—Checklist of items that should be included in reports of cohort studies

|  | **Item No** | **Recommendation** | **Reported on Page No.** |
| --- | --- | --- | --- |
| **Title and abstract** | 1 | (*a*) Indicate the study’s design with a commonly used term in the title or the abstract | Title |
|  |  | (*b*) Provide in the abstract an informative and balanced summary of what was done and what was found | Abstract – pp 1 |
| **Introduction** | | |  |
| Background/rationale | 2 | Explain the scientific background and rationale for the investigation being reported | Introduction – pp 4, paragraph 3 |
| Objectives | 3 | State specific objectives, including any prespecified hypotheses | Introduction – pp 4 paragraph 4 |
| **Methods** | | |  |
| Study design | 4 | Present key elements of study design early in the paper | Methods – pp 5,  paragraph 3 |
| Setting | 5 | Describe the setting, locations, and relevant dates, including periods of recruitment, exposure, follow-up, and data collection | Methods – pp 5, |
| Participants | 6 | (*a*) Give the eligibility criteria, and the sources and methods of selection of participants. Describe methods of follow-up  (*b*) For matched studies, give matching criteria and number of exposed and unexposed | Methods – pp 5,  Supplementary file - Figure S2  N/A |
| Variables | 7 | Clearly define all outcomes, exposures, predictors, potential confounders, and effect modifiers. Give diagnostic criteria, if applicable | Methods – pp 5-7 |
| Data sources/ measurement | 8* | For each variable of interest, give sources of data and details of methods of assessment (measurement). Describe comparability of assessment methods if there is more than one group | Methods – pp 6, paragraph 1 |
| Bias | 9 | Describe any efforts to address potential sources of bias | Methods - pp 7, paragraph 2 |
| Study size | 10 | Explain how the study size was arrived at | Methods - pp 5,  Paragraph 2,  Supplementary figure S2 |
| Quantitative variables | 11 | Explain how quantitative variables were handled in the analyses. If applicable, describe which groupings were chosen and why | Methods - pp 6-7, paragraphs 2-3 |
| Statistical methods | 12 | (*a*) Describe all statistical methods, including those used to control for confounding | Methods – pp 7, paragraphs 2 |
|  |  | (*b*) Describe any methods used to examine subgroups and interactions | Methods - pp 7, paragraph 2 |
|  |  | (*c*) Explain how missing data were addressed | Methods - pp 7, paragraph 2 |
|  |  | (*d*) If applicable, describe analytical methods taking account of sampling strategy | Methods - pp 7, paragraph 3 |
|  |  | (*e*) Describe any sensitivity analyses | Methods - pp 7, paragraph 2 |
| **Results** | | |  |
| Participants | 13* | (a) Report numbers of individuals at each stage of study—eg numbers potentially eligible, examined for eligibility, confirmed eligible, included in the study, completing follow-up, and analysed | Results – pp 7, paragraph 2 |
|  |  | (b) Give reasons for non-participation at each stage | Supplementary Figure S2 |
|  |  | (c) Consider use of a flow diagram | Supplementary Figure S2 |
| Descriptive data | 14* | (a) Give characteristics of study participants (eg demographic, clinical, social) and information on exposures and potential confounders | Results – pp 8 paragraph 2, Table 1 |
|  |  | (b) Indicate number of participants with missing data for each variable of interest  (c) Summarise follow-up time (eg, average and total amount) | Supplementary Figure S2 |
| Outcome data | 15* | Report numbers of outcome events or summary measures | Table 1 |
| Main results | 16 | (*a*) Give unadjusted estimates and, if applicable, confounder-adjusted estimates and their precision (eg, 95% confidence interval). Make clear which confounders were adjusted for and why they were included | Results -pp 9, paragraph 3 |
|  |  | (*b*) Report category boundaries when continuous variables were categorized | N/A |
|  |  | (*c*) If relevant, consider translating estimates of relative risk into absolute risk for a meaningful time period | N/A |
| Other analyses | 17 | Report other analyses done—eg analyses of subgroups and interactions, and sensitivity analyses | Results – pp 8, paragraph 2 |
| **Discussion** | | |  |
| Key results | 18 | Summarise key results with reference to study objectives | Discussion – pp 11 paragraphs 3 |
| Limitations | 19 | Discuss limitations of the study, taking into account sources of potential bias or imprecision. Discuss both direction and magnitude of any potential bias | Discussion – pp 13, paragraph 3 |
| Interpretation | 20 | Give a cautious overall interpretation of results considering objectives, limitations, multiplicity of analyses, results from similar studies, and other relevant evidence | Discussion – pp 13 paragraphs 3 |
| Generalisability | 21 | Discuss the generalisability (external validity) of the study results | Discussion – pp 14 paragraph 3 |
| **Other information** | | |  |
| Funding | 22 | Give the source of funding and the role of the funders for the present study and, if applicable, for the original study on which the present article is based | pp 1 |

*Give information separately for exposed and unexposed groups.

**Note:** An Explanation and Elaboration article discusses each checklist item and gives methodological background and published examples of transparent reporting. The STROBE checklist is best used in conjunction with this article (freely available on the Web sites of BMJ Open at https://www.equator-network.org/reporting-guidelines/strobe/. Information on the STROBE Initiative is available at [www.strobe-statement.org](http://www.strobe-statement.org).
