# Supplementary material for: Childhood socio-emotional and cognitive development and adolescents NEET (not in education, employment or training): findings from the UK Millennium Cohort Study
Source: BMJ Open. 2026 Jun 22;16(6):e109720. doi: 10.1136/bmjopen-2025-109720 (PMC13288871; doi:10.1136/bmjopen-2025-109720)
Supplement: Supplementary file 2 [file bmjopen-2025-109720.reviewer_comments.pdf]

PEER REVIEW HISTORY

BMJ Open publishes all reviews undertaken for accepted manuscripts. Reviewers are asked to complete a checklist review form and are provided with free text boxes to elaborate on their assessment. These free text comments are reproduced below.

ARTICLE DETAILS

Title (Provisional)

Childhood socioemotional and cognitive development and Adolescents NEET (not in education, employment or training): findings from the UK Millennium Cohort Study

Authors

Akanni, Lateef; Black, Michelle; Udu, Kalu; Chen, Yanhua; Cattermore, Rosalie; Esan, Oluwaseun B; Creese, Hanna; Melendez-Torres, G.J; Hargreaves, Dougal S; Adjei, N; Taylor-Robinson, David

---

VERSION 1 - REVIEW

Reviewer 1

Name Inagawa, Takuma

Affiliation National Center of Neurology and Psychiatry, National Center of Neurology

Date 14-Nov-2025

COI None

Thank you for giving me the opportunity to review this manuscript.

1) the authors did not assess the null hypothesis by using p-values at all.

2) No checklist has been attached.

Therefore, it is impossible to assess the research quality rigorously. I think the authors must resubmit the article after planning statistical analysis more rigorously, conducting statistical analysis, and attaching any checklist like STROBE.

Reviewer 2

Name Austin, Topun

Affiliation University of Cambridge

Date 25-Jan-2026

COI None

This paper examines how childhood cognitive and socioemotional development trajectories from ages 3-14 years relate to NEET (not in education, employment, or training) status at age 17 in the UK Millennium Cohort Study.

The researchers analyzed data from 8,368 children, identifying four developmental trajectory groups: no problems (76.5%), early and resolving problems (8.6%), late socioemotional problems (10.1%), and persistent cognitive and socioemotional problems (4.8%). At age 17, 3.5% of participants were NEET, with 38% economically inactive.

Key findings showed that children with persistent cognitive and socioemotional problems had 3.5-fold increased NEET risk (adjusted risk ratio 3.52, 95% CI 2.31-5.34), while those with late socioemotional problems had 3-fold increased risk (3.00, 95% CI 2.08-4.32). Notably, early problems that resolved were not significantly associated with NEET status. The authors estimated that approximately 28% of NEET cases could be attributed to childhood developmental problems, rising to 34% for economically inactive NEET.

The study concludes that interventions targeting cognitive and socioemotional skills in early childhood, particularly in the first years of primary school, may effectively reduce NEET prevalence.

This study makes an important contribution to understanding the developmental origins of youth disengagement from education and employment. The use of a large longitudinal cohort and the use of trajectory modelling to capture the temporal dynamics of development is a real methodological strength that moves beyond simple cross-sectional associations. The finding that early, resolving problems don't increase NEET risk while late-emerging or persistent problems do has significant policy implications, suggesting critical windows for intervention.

However, there are several limitations some of which are addressed by the authors. First, the single-timepoint measurement of NEET status at age 17 is always going to be problematic. NEET is known to be a fluid state with frequent transitions, and capturing status at one moment may misclassify trajectories. Some youth classified as NEET may be in temporary gaps between education or employment, while others may experience chronic disengagement. This heterogeneity matters for both understanding mechanisms and targeting interventions.

Second, while the authors adjust for key confounders, the causal interpretation implicit in the population attributable fraction calculations is questionable. The DAG presented is relatively simple and may not adequately capture the complex, bidirectional relationships between socioeconomic disadvantage, family adversity, child development, and educational/employment outcomes. Unmeasured confounding—particularly regarding family mental health, parenting quality, and neighbourhood effects—likely remains.

Third, the mechanisms linking developmental trajectories to NEET status are underexplored. The authors suggest that resolving socioemotional problems may be "protective" despite

poor educational attainment, but this raises questions: What drives this protection? Is it improved peer relationships, teacher perceptions, self-efficacy, or something else? Understanding mechanisms would strengthen intervention design.

The study's generalizability is also uncertain. As with all longitudinal cohort studies there is a temporal delay - the cohort was born in 2000-2002, and the current policy and economic landscape—including post-pandemic effects on youth mental health and employment—may differ substantially. Additionally, attrition in longitudinal studies tends to be socially patterned, potentially underrepresenting the most disadvantaged youth most at risk of NEET.

As mentioned, the authors do address most of these limitations, however some comment on the effect of the pandemic on future trajectories may be helpful.

In conclusion the paper provides valuable evidence that early socioemotional development matters for later economic participation beyond academic achievement alone. This supports integrated approaches addressing both cognitive and non-cognitive skills, and challenges purely test-focused educational policies. As such this is an important paper, not just for its scientific content but to inform public policy.

**Reviewer**	**3**
**Name**	**Richards, Marcus**
**Affiliation**	**University College London**
**Date**	**18-Feb-2026**
**COI**	**None**

In this observational study using the UK Millennium Cohort Study, the authors report that, assuming causality, approximately 28% of NEET cases (not in education, employment or training) at age 17 years were attributable to cognitive and socioemotional problems in childhood. While early and resolving problems were not associated with this outcome, there was high risk in those with later and, especially, persistent problems. Furthermore, this risk pattern was more severe in those who were NEET and economically inactive (not looking for work) compared to those who were NEET but actively seeking work. Analyses were adjusted for sex, maternal education and ethnicity, and income; robustness of group trajectory membership was tested; and analyses accounted for missing covariable data.

General comments

While the overall topic has been well-investigated, the findings in relation to whether participants were actively seeking work are an interesting contribution. It is a pity, therefore, that the authors skim over this aspect, both regarding interpretation and implications for potentially differential intervention. Instead, the bulk of the Discussion is devoted to more

generalised implications for intervention, which have long been recommended for targeting such developmental problems.

Regarding context, it is interesting to compare these findings with a related study using the British 1946, 1958 and 1970 birth cohorts (http://www.centreformentalhealth.org.uk/pdfs/life_chances_summary.pdfhttp://www.centreformentalhealth.org.uk/pdfs/life_chances_report.pdf). Here, adolescent emotional problems were divided into mild and severe; were adjusted for childhood cognition and conduct problems since the latter tend to overlap; and educational and employment outcomes were disaggregated. In crude summary, odds of no educational qualifications and chronic economic inactivity were – unsurprisingly - elevated in those with severe emotional problems compared to those with mild emotional problems.

Specific comments

It is not consistently correct to say that adjustment for covariables had “minimal impact” on the strength of associations (page 10 line 36). Table 2 shows risk attenuation by covariable adjustment in those with persistent problems from 6.28 to 3.52 for NEET and looking for work, and 9.44 to 6.29 for NEET and not looking for work, reductions that hardly seem trivial.

Regarding the covariables themselves (sex, maternal education and ethnicity, and income), it would be interesting to know the rationale for their selection, and whether any of these differentially accounted for risk attenuation. I would imagine that this is income in particular and wonder how the authors interpret the residual aspects of NEET after this is controlled.

VERSION 1 - AUTHOR RESPONSE

Reviewer 1 Comments

The authors did not assess the null hypothesis by using p-values at all. No checklist has been attached. Therefore, it is impossible to assess the research quality rigorously. I think the authors must resubmit the article after planning statistical analysis more rigorously, conducting statistical analysis, and attaching any checklist like STROBE.

Response: We thank the reviewer you for this comment. We did not rely on p-values; instead, we used confidence intervals to assess the estimates. Confidence intervals provide information about both the magnitude and precision of the estimated association and increasingly used and the common reporting standards in public health research. We agree that clear reporting of methods is essential for rigorous appraisal. We have therefore revised the manuscript to further clarify our statistical analysis and its rationale. In addition, we have now included a completed STROBE checklist to support transparent evaluation of the study.

Page 6: “All estimates were reported with 95% confidence intervals which provides information about both the magnitude and precision of the estimated association.”

Page 4: “This study is reported in accordance with the STROBE guidelines (see Supplementary Table S2).”

Supplementary file – Table S2

Checklist. STROBE Statement—Checklist of items that should be included in reports of cohort studies

	Item No	Recommendation	Reported on Page No.
**Title and abstract**	1	(*a*) Indicate the study’s design with a commonly used term in the title or the abstract	Title
		(*b*) Provide in the abstract an informative and balanced summary of what was done and what was found	Abstract – pp 1
**Introduction**			
Background/rationale	2	Explain the scientific background and rationale for the investigation being reported	Introduction – pp 4, paragraph 3
Objectives	3	State specific objectives, including any prespecified hypotheses	Introduction – pp 4 paragraph 4
**Methods**			
Study design	4	Present key elements of study design early in the paper	Methods – pp 5, paragraph 3
Setting	5	Describe the setting, locations, and relevant dates, including periods of recruitment, exposure, follow-up, and data collection	Methods – pp 5,
Participants	6	(*a*) Give the eligibility criteria, and the sources and methods of selection of participants. Describe methods of follow-up	Methods – pp 5, Supplementary file - Figure S2
		(*b*) For matched studies, give matching criteria and number of exposed and unexposed	N/A
Variables	7	Clearly define all outcomes, exposures, predictors, potential confounders, and effect modifiers. Give diagnostic criteria, if applicable	Methods – pp 5-7
Data sources/ measurement	8*	For each variable of interest, give sources of data and details of methods of assessment (measurement). Describe comparability of assessment methods if there is more than one group	Methods – pp 6, paragraph 1
Bias	9	Describe any efforts to address potential sources of bias	Methods - pp 7, paragraph 2

Study size	10	Explain how the study size was arrived at	Methods - pp 5, Paragraph 2, Supplementary figure S2
Quantitative variables	11	Explain how quantitative variables were handled in the analyses. If applicable, describe which groupings were chosen and why	Methods - pp 6-7, paragraphs 2-3
Statistical methods	12	(*a*) Describe all statistical methods, including those used to control for confounding	Methods – pp 7, paragraphs 2
		(*b*) Describe any methods used to examine subgroups and interactions	Methods - pp 7, paragraph 2
		(*c*) Explain how missing data were addressed	Methods - pp 7, paragraph 2
		(*d*) If applicable, describe analytical methods taking account of sampling strategy	Methods - pp 7, paragraph 3
		(*e*) Describe any sensitivity analyses	Methods - pp 7, paragraph 2
**Results**			
Participants	13*	(a) Report numbers of individuals at each stage of study—eg numbers potentially eligible, examined for eligibility, confirmed eligible, included in the study, completing follow-up, and analysed	Results – pp 7, paragraph 2
		(b) Give reasons for non-participation at each stage	Supplementary Figure S2
		(c) Consider use of a flow diagram	Supplementary Figure S2
Descriptive data	14*	(a) Give characteristics of study participants (eg demographic, clinical, social) and information on exposures and potential confounders	Results – pp 8 paragraph 2, Table 1
		(b) Indicate number of participants with missing data for each variable of interest<br>(c) Summarise follow-up time (eg, average and total amount)	Supplementary Figure S2
Outcome data	15*	Report numbers of outcome events or summary measures	Table 1
Main results	16	(*a*) Give unadjusted estimates and, if applicable, confounder-adjusted estimates and their precision (eg, 95% confidence interval). Make clear which confounders were adjusted for and why they were included	Results -pp 9, paragraph 3
		(*b*) Report category boundaries when continuous variables were categorized	N/A
		(*c*) If relevant, consider translating estimates of relative risk into absolute risk for a meaningful time period	N/A

Other analyses	17	Report other analyses done—eg analyses of subgroups and interactions, and sensitivity analyses	Results – pp 8, paragraph 2
**Discussion**			
Key results	18	Summarise key results with reference to study objectives	Discussion – pp 11 paragraphs 3
Limitations	19	Discuss limitations of the study, taking into account sources of potential bias or imprecision. Discuss both direction and magnitude of any potential bias	Discussion – pp 13, paragraph 3
Interpretation	20	Give a cautious overall interpretation of results considering objectives, limitations, multiplicity of analyses, results from similar studies, and other relevant evidence	Discussion – pp 13 paragraphs 3
Generalisability	21	Discuss the generalisability (external validity) of the study results	Discussion – pp 14 paragraph 3
**Other information**			
Funding	22	Give the source of funding and the role of the funders for the present study and, if applicable, for the original study on which the present article is based	pp 1

*Give information separately for exposed and unexposed groups.
Note: An Explanation and Elaboration article discusses each checklist item and gives methodological background and published examples of transparent reporting. The STROBE checklist is best used in conjunction with this article (freely available on the Web sites of BMJ Open at https://www.equator-network.org/reporting-guidelines/strobe/. Information on the STROBE Initiative is available at www.strobe-statement.org.

Reviewer 2 Comments

First, the single-timepoint measurement of NEET status at age 17 is always going to be problematic. NEET is known to be a fluid state with frequent transitions, and capturing status at one moment may misclassify trajectories. Some youth classified as NEET may be in temporary gaps between education or employment, while others may experience chronic disengagement. This heterogeneity matters for both understanding mechanisms and targeting interventions.

Response: We thank the Reviewer for the comment. We agree that NEET is a dynamic state and that measurement at a single time point cannot distinguish between temporary and longer-term disengagement. We have revised the manuscript to make this limitation clearer. However, age 17 remains a substantively important stage, as it captures a key transition between compulsory education and early labour market engagement. The aim of this study was to examine whether childhood cognitive and socioemotional developmental trajectories are associated with risk of being NEET at this critical transition point, rather than to characterise longer-term NEET patterns. We now state this more explicitly in the discussion

and note that future research using repeated longitudinal measures of NEET would help distinguish temporary from persistent disengagement.

Page 13: “The study has some limitations. NEET status was measured at a single time point, which may not capture the dynamic nature of youth disengagement or transitions from compulsory education into further education, training, or employment. Although this is a common limitation (Elsenburg et al., 2025), NEET status at age 17 is of particular relevance given the potential long-term implications for health and employment (Guido, 2015). Future studies using longitudinal and repeated measures of NEET are needed to distinguish temporary from longer-term disengagement.”

Second, while the authors adjust for key confounders, the causal interpretation implicit in the population attributable fraction calculations is questionable. The DAG presented is relatively simple and may not adequately capture the complex, bidirectional relationships between socioeconomic disadvantage, family adversity, child development, and educational/employment outcomes. Unmeasured confounding—particularly regarding family mental health, parenting quality, and neighbourhood effects—likely remains.

Response: Thank you. To explain the unmeasured confounding between NEET and the developmental trajectories, we have now computed E-value as a sensitivity analysis. We used the E-value, introduced by VanderWeele and Ding (2017), to show the minimum strength of unmeasured confounding and explain away the association between child developmental trajectories (exposure) and NEET status (outcome) beyond the measured confounders included in our analysis. The results are discussed in the revised manuscripts and included an in the appendix.

Page 9: “Additionally, the E-values indicated that the estimated associations for late and persistent cognitive and socioemotional problems were robust to unmeasured confounding but not for early and resolving cognitive and socioemotional problems (See supplementary file – Table S1).”

Supplementary appendix –
Table S1: E-values for unmeasured confounders

Ref: *No problems*	OR	RR E-value	CI E-value
Early and resolving cognitive and socioemotional problems	1.29	1.93	1.00
Late socioemotional problems	3.00	5.98	3.81
Persistent cognitive and socioemotional problems	3.52	7.38	4.38

Note: The Table provides the calculated E-values to assess the robustness of observed associations to potential unmeasured confounding. As NEET prevalence was below 10%, odds ratios were interpreted as approximations to risk ratios when deriving E-values. The associations for late socioemotional and persistent cognitive and socioemotional problems show that relatively strong unmeasured confounding would be required to fully explain the observed estimates. In contrast, the association for early and resolving problems showed limited robustness, suggesting that weaker unmeasured confounding could account for the observed association.

Third, the mechanisms linking developmental trajectories to NEET status are underexplored. The authors suggest that resolving socioemotional problems may be "protective" despite poor

educational attainment, but this raises questions: What drives this protection? Is it improved peer relationships, teacher perceptions, self-efficacy, or something else? Understanding mechanisms would strengthen intervention design.

Response: Thank you for highlighting this important point and we agree that it warrants more attention. We have added to the relevant part of the discussion.

Page 11: "Further research could help to unpick these protective mechanisms building on the mediating mechanism of socialization through peers, schools and family to better understand how social and emotional skills in childhood forecast skills in late adolescent/adulthood for labour market success (Del Bono et al 2024)."

The study's generalizability is also uncertain. As with all longitudinal cohort studies there is a temporal delay - the cohort was born in 2000-2002, and the current policy and economic landscape—including post-pandemic effects on youth mental health and employment—may differ substantially. Additionally, attrition in longitudinal studies tends to be socially patterned, potentially underrepresenting the most disadvantaged youth most at risk of NEET.

Response: Thank you. We have addressed the issue of policy relevance and the pandemic in the limitations section. Also, we acknowledged the attrition risks in the study limitation and clarified that this could result in underestimation of the observed associations.

Page 13: "As with any longitudinal study the temporal delay between the analysis of the cohort (age 17 in 2017) and the present day may limit what we can infer for policy relevance. Whilst we have shown that the prevalence of NEET in 2025 is similar to that of 2017, in that time we have had a global pandemic with lasting impact on the mental health and wellbeing of young people which may mean that policy interventions to reduce the number of young people who are NEET today, may need to have a specific focus on mental health. Finally, attrition and missingness are major concerns in longitudinal studies as they pose threats to data quality and could result in estimate bias and underestimation of the observed associations."

As mentioned, the authors do address most of these limitations, however some comment on the effect of the pandemic on future trajectories may be helpful.

Response: Thank you. This has now been added as per the response to the previous comment.

Page 13: "As with any longitudinal study the temporal delay between the analysis of the cohort (age 17 in 2017) and the present day may limit what we can infer for policy relevance. Whilst we have shown that the prevalence of NEET in 2025 is similar to that of 2017, in that time we have had a global pandemic with lasting impact on the mental health and wellbeing of young people which may mean that policy interventions to reduce the number of young people who are NEET today, may need to have a specific focus on mental health. "

Reviewer 3 Comments

While the overall topic has been well-investigated, the findings in relation to whether participants were actively seeking work are an interesting contribution. It is a pity, therefore,

that the authors skim over this aspect, both regarding interpretation and implications for potentially differential intervention. Instead, the bulk of the Discussion is devoted to more generalised implications for intervention, which have long been recommended for targeting such developmental problems.

Regarding context, it is interesting to compare these findings with a related study using the British 1946, 1958 and 1970 birth cohorts (http://www.centreformentalhealth.org.uk/pdfs/life_chances_summary.pdfhttp://www.centreformentalhealth.org.uk/pdfs/life_chances_report.pdf). Here, adolescent emotional problems were divided into mild and severe; were adjusted for childhood cognition and conduct problems since the latter tend to overlap; and educational and employment outcomes were disaggregated. In crude summary, odds of no educational qualifications and chronic economic inactivity were – unsurprisingly - elevated in those with severe emotional problems compared to those with mild emotional problems.

Response: Thank you for highlighting these interesting reports. We agree that they are helpful for our discussion, particularly in relation to conduct and emotional problems (both measures within SDQ, our measure of socioemotional behaviour problems). We have added a section to the relevant part of the discussion.

Pages 11-12: "In relation to the development of socioemotional behaviour problems in late childhood/early adolescence we see over a threefold increase in the risk of both NEET status and being NEET and not economically active. This reflects findings in relation to conduct and emotional problems which have been shown in UK cohorts, from 1958 and 1970, to be associated with increased odds of economic inactivity, moreso for conduct problems (refs). Understanding how to better support these aspects of development are crucial if we are to address the increasing prevalence of economic inactivity in working age people"

It is not consistently correct to say that adjustment for covariables had "minimal impact" on the strength of associations (page 10 line 36). Table 2 shows risk attenuation by covariable adjustment in those with persistent problems from 6.28 to 3.52 for NEET and looking for work, and 9.44 to 6.29 for NEET and not looking for work, reductions that hardly seem trivial.

Regarding the covariables themselves (sex, maternal education and ethnicity, and income), it would be interesting to know the rationale for their selection, and whether any of these differentially accounted for risk attenuation. I would imagine that this is income in particular and wonder how the authors interpret the residual aspects of NEET after this is controlled.

Response: Thank you. We have adjusted the results section to discuss the estimates attenuation after sequential adjustment of each confounder.

Page 8: "Estimates were attenuated after adjusting for confounders. However, sequential adjustment for each confounders indicated that income quintile at baseline accounted for the largest proportion of the association between NEET status and developmental trajectories, with maternal education, an indication of early life socioeconomic conditions contributing

further. The residual association after controlling for these early-life socioeconomic factors suggests that NEET status at adolescent captures additional risk not explained by early-life disadvantage. (See Supplementary file – Figure S6).”

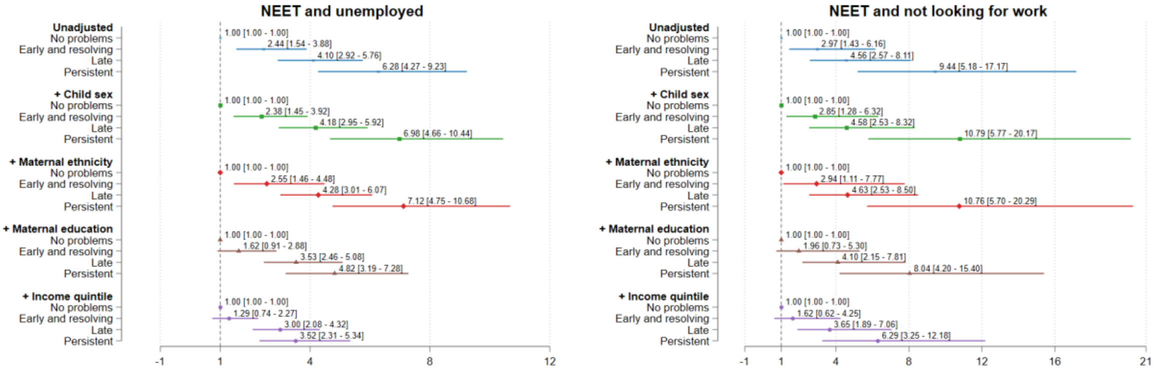

Figure S 1: Sequential adjustment for confounders

VERSION 2 - REVIEW

**Reviewer**	**3**
**Name**	**Richards, Marcus**
**Affiliation**	**University College London**
**Date**	**02-Apr-2026**
**COI**	

I am satisfied with the authors' revisions to this manuscript.
